# Immunological Aspects of Chytridiomycosis

**DOI:** 10.3390/jof6040234

**Published:** 2020-10-19

**Authors:** Laura F. Grogan, Josephine E. Humphries, Jacques Robert, Chantal M. Lanctôt, Catherine J. Nock, David A. Newell, Hamish I. McCallum

**Affiliations:** 1 Environmental Futures Research Institute and School of Environment and Science, Griffith University, Southport, QLD 4222, Australia; h.mccallum@griffith.edu.au; 2 Forest Research Centre, School of Environment, Science and Engineering, Southern Cross University, Lismore, NSW 2480, Australia; j.humphries.17@student.scu.edu.au (J.E.H.); David.Newell@scu.edu.au (D.A.N.); 3 University of Rochester Medical Center, Rochester, NY 14642, USA; Jacques_Robert@urmc.rochester.edu; 4 Australian Rivers Institute, Griffith University, Southport, QLD 4222, Australia; c.lanctot@griffith.edu.au; 5 Southern Cross Plant Science, Southern Cross University, Lismore, NSW 2480, Australia; cathy.nock@scu.edu.au

**Keywords:** chytridiomycosis, *Batrachochytrium dendrobatidis*, *Batrachochytrium salamandrivorans*, amphibian, disease, immunopathology, immunosuppression, innate, adaptive, constitutive

## Abstract

Amphibians are currently the most threatened vertebrate class, with the disease chytridiomycosis being a major contributor to their global declines. Chytridiomycosis is a frequently fatal skin disease caused by the fungal pathogens *Batrachochytrium dendrobatidis* (Bd) and *Batrachochytrium salamandrivorans* (Bsal). The severity and extent of the impact of the infection caused by these pathogens across modern Amphibia are unprecedented in the history of vertebrate infectious diseases. The immune system of amphibians is thought to be largely similar to that of other jawed vertebrates, such as mammals. However, amphibian hosts are both ectothermic and water-dependent, which are characteristics favouring fungal proliferation. Although amphibians possess robust constitutive host defences, Bd/Bsal replicate within host cells once these defences have been breached. Intracellular fungal localisation may contribute to evasion of the induced innate immune response. Increasing evidence suggests that once the innate defences are surpassed, fungal virulence factors suppress the targeted adaptive immune responses whilst promoting an ineffectual inflammatory cascade, resulting in immunopathology and systemic metabolic disruption. Thus, although infections are contained within the integument, crucial homeostatic processes become compromised, leading to mortality. In this paper, we present an integrated synthesis of amphibian post-metamorphic immunological responses and the corresponding outcomes of infection with Bd, focusing on recent developments within the field and highlighting future directions.

## 1. Introduction

Amphibians are the most threatened vertebrate class [1]. A range of factors has contributed to their declines, including habitat degradation, invasive species, climate change and emerging infectious diseases [2]. In particular, the fungal disease chytridiomycosis has been a major cause of global amphibian declines over the last several decades [3]. Chytridiomycosis is a frequently fatal skin disease of amphibians that is caused by the fungal pathogens *Batrachochytrium dendrobatidis* (Bd) and the more recently discovered *Batrachochytrium salamandrivorans* (Bsal) [4,5,6]. Importantly, the severity and extent of the impact of the infection caused by these pathogens across the modern amphibian groups are unprecedented in the history of vertebrate infectious diseases [7] (see Box 1 for an overview of chytridiomycosis). Effective mitigation strategies for declining wild populations remain elusive [8], although recent evidence suggests that evolutionary changes in the host immune response can occur rapidly in some species, favouring recovery from the threat of Bd [9] (see Box 2).

Box 1 **Chytridiomycosis—impact, taxonomy, host range and disease.**

**Figure 1:**
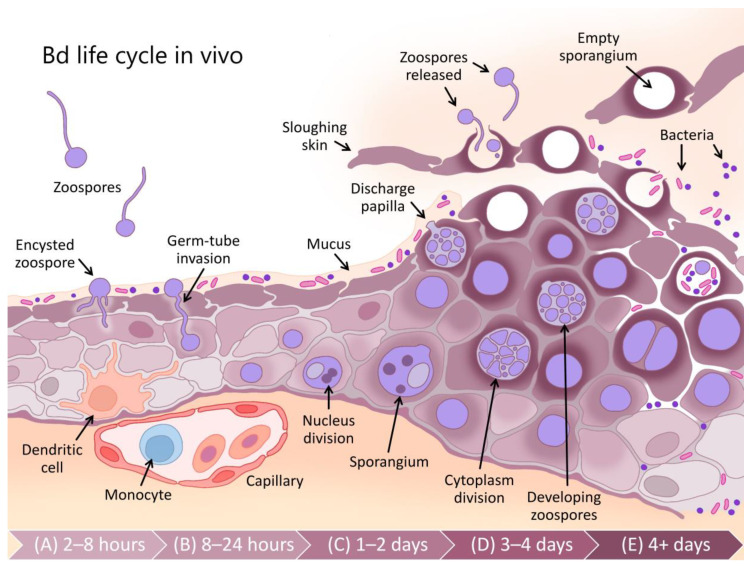
Schematic of the in vivo life cycle of *Batrachochytrium dendrobatidis* (Bd) depicting a microscopic cross-section of amphibian skin (cellular epidermis) and the process of Bd invasion, replication and release. Approximate times/stages during the initial infection process are depicted from left to right (**A**–**E**). Bd is depicted in violet and host skin cells are depicted in mauve, with increasing cell keratinisation being darker. During the first 2–8 h post exposure (**A**), free-living infectious zoospores undergo chemotaxis towards the skin surface using their motile flagellum, where they encyst into a thallus, absorb the flagellum and develop rhizoids. From there (**B**), they produce a germ tube through which they inject their contents into deep cells of the host epidermis. From 1–2 days post exposure (**C**), the developing sporangium undergoes division of its nucleus, then cytoplasm, yielding individual zoospores which develop inside the sporangial capsule. These zoospores are then released through a discharge tube (**D**). Note that the infected skin demonstrates cellular hypertrophy (larger cells), epidermal hyperplasia (increased thickness due to an increase in cell number), hyperkeratosis (increased cellular keratin shown in darker mauve), and skin sloughing becomes dysregulated. Continued infection (**E**) disrupts the integrity of the epithelial barrier, permitting secondary bacterial infection.

Chytridiomycosis is a frequently fatal fungal skin disease that is thought to be responsible for declines in at least 501 amphibian species, 90 of which are presumed extinct [3,7]. The disease is caused by two pathogenic fungi (*Batrachochytrium dendrobatidis* (Bd) and *B. salamandrivorans* (Bsal) [4,5,6]) of the phylum Chytridiomycota [10]. The Chytridiomycota are ubiquitous aerobic saprotrophs (utilising predominantly chitin and keratin) in water and soil habitats. They are characterised by a diphasic life cycle comprising motile uniflagellate zoospores and a reproductive thallus (known as a zoosporangium) resembling a pot-like shape (hence the Greek χυτρίδιον (chytridion), or pyre vessel [11]; Figure 1). Although many chytrids parasitise plants, algae and other fungi, Bd and Bsal are remarkable as pathogens of vertebrates [12].

Bd is now found in amphibians around the world [13] but originated from southeast Asia [14]. It has a wide host range, including frogs, salamanders and caecilians, but is most virulent in frogs [15,16]. Bsal is known from outbreaks in European salamanders, has a similar ancestral range to Bd, but has not yet been detected in North America, although its introduction is likely [6,14,17,18]. While it infects salamanders and frogs, it does not appear to produce large-scale mortality in frogs, possibly due to its lower thermal preference (5–25 °C, with optimal growth at 10–15 °C) [6,16]. However, co-infections with Bd and Bsal are possible and may contribute to increasing mortality [19,20]. This review concentrates primarily on Bd, as much more is known about its immunology and pathology (although, see [6,19,20,21,22,23]).

Bd and Bsal are intracellular pathogens of amphibian skin (see Figure 1). The motile zoospores of Bd target the keratinised epidermal cells of amphibians [24], including the entire skin surface of post-metamorphic adults and the keratinised mouthparts of tadpoles [25]. As an infection develops within the skin, histopathological changes due to Bd include hyperkeratosis, hyperplasia, ulceration and erosion [24,26]. In contrast, infection with Bsal causes erosion, ulceration and necrosis, without evidence of hyperplasia or hyperkeratosis [16]. Continued infection with Bd can lead to the disruption of normal skin functioning, systemic metabolic dysregulation, electrolyte imbalance and subsequent cardiac arrest [27,28,29]. Clinical signs of late-stage Bd infection include increased skin shedding, lethargy, inappetence and loss of the righting reflex [26]. Fire salamanders (*Salamandra salamandra*) infected with Bsal showed anorexia, lethargy and ataxia, with infections frequently resulting in rapid mortality [6]. Mortality rates up to 100% due to Bd have been observed in some species [4].

Box 2 **Chytridiomycosis—an evolutionary perspective.**

The early 20th-century emergence of the fungal pathogen *Batrachochytrium dendrobatidis* (Bd) has been traced to the Korean peninsula [14]. Several Bd lineages with differing levels of pathogenicity have been identified [14,30]. The hypervirulent Bd lineage BdGPL is reportedly responsible for pandemic chytridiomycosis and global declines in amphibian biodiversity [31]. As the centre of origin, East Asia has the greatest Bd diversity. Asian amphibian species appear to be more resilient to Bd infection; they less frequently display clinical signs and no mass mortalities have been reported to date [32,33]. There is little evidence for the attenuation of virulence of BdGPL since its emergence and spread [34]. This is likely due to the presence of biological pathogen reservoirs that permit Bd persistence despite significant declines in hosts [35,36,37,38].

In contrast, there is growing evidence for evolutionary change in the host immune response. A number of amphibian species that initially severely declined due to Bd now appear to be recovering [3,39]. Indeed, some species previously thought to be extinct have since been rediscovered [40]. Recent immunogenetic evidence suggests that evolutionary changes in the host favouring immune resistance at the major histocompatibility complex (MHC) locus can occur rapidly in some species [9]. Other genes associated with constitutive defences (such as antimicrobial peptides) may also be involved [34]. Unfortunately, many amphibian species have already been extirpated without any evidence of recovery or reappearance, and many more species are still declining [3].

The immune system of adult amphibians is thought to be fundamentally conserved in structure and function with respect to other jawed vertebrates, such as mammals, despite their early evolutionary divergence. Indeed, amphibians, particularly *Xenopus* spp. have frequently been used as a model for human medical immunology [41,42] due to their experimental suitability and perceived similarities between the *Xenopus laevis* immune system and that of mammals [43,44]. As a result, although amphibians are ecologically significant in their own right, understanding amphibian immunological responses may also be of value with respect to human medicine [42,45]. Nevertheless, there are some important distinctions between amphibian and mammalian immune systems [44]. Structurally, the lymph nodes of mammals are absent from most amphibians, and instead, lymphopoiesis, antigen presentation and lymphocyte expansion occur predominantly in the spleen [46]. The liver, thymus, kidneys and intestine are also immunologically important sites in amphibians [47]. Although the innate immune system is similar, compared with mammals, amphibians possess a greatly expanded antimicrobial peptide (AMP) repertoire that is more diverse than that of any other vertebrate group studied thus far [48,49]. Regarding the adaptive immune response, amphibians possess a reduced lymphocyte assemblage [43], their lymphocytes demonstrate poorer affinity maturation [50] and their cytotoxic T cell expansion is less extensive compared with mammals [51]. Interestingly, unlike in mammals, mature amphibian B cells can perform phagocytosis [52]. Amphibian and mammalian immunoglobulins are structurally and functionally similar, with IgM and IgD being present in both groups, and IgX and IgY of amphibians being functionally analogous to the IgA and IgG of mammals, although amphibian IgF does not have a known mammalian homologue [53,54,55,56].

Despite possessing a robust immune system, two key characteristics of amphibian life histories that vary from mammals promote fungal proliferation and could be considered to increase amphibian vulnerability to fungal pathogens. First, amphibian hosts are ectothermic, regulating their body temperature through behaviour and exposure to external sources of heat, such as sunlight and warm surface environments, infrequently achieving temperatures as high as 35 °C [57] (see Box 3 for an overview of the importance of amphibian ectothermy for infection). Correspondingly, most fungi are mesophiles, preferring moderate temperatures below 35 °C [58]. In contrast, the high body temperatures of endotherms (35–40 °C), such as mammals and birds, are thought to act as a non-specific defence against fungal infections [59]. Second, amphibians are typically heavily reliant on water, with at least part of their lifecycle tied to fresh (or, less commonly, brackish) water [60]. Many amphibian species thus inhabit environments such as rainforest streams that are also favoured by saprophytic fungi that rely on external energy sources from decomposing organic matter [61]. For example, Bd and Bsal originated from the ancient phylum Chytridiomycota, which contains predominantly saprophytic species [21,62]. However, excepting chytridiomycosis, reports of amphibian vulnerability to fungal pathogens are limited (although, see [26,63,64]), which is likely due to their expanded and highly efficacious constitutive (always present) defence system, particularly the AMPs [48,49].

Box 3 **Amphibian ectothermy and chytridiomycosis.**

**Figure 2:**
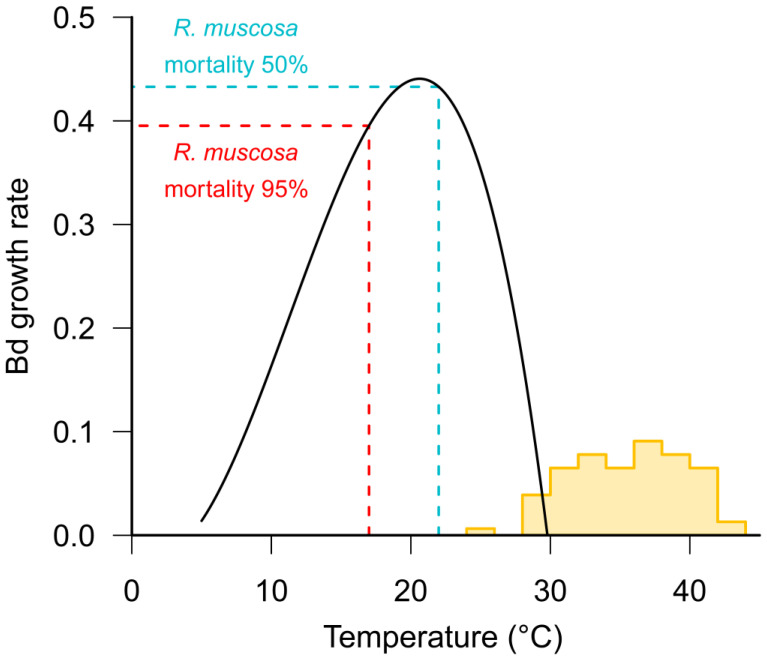
Effects of temperature on *Batrachochytrium dendrobatidis* (Bd) growth rates and amphibian survival. The Bd growth rate (black line) from Piotrowski et al. [65], using a curve fitted by Rohr et al. [81], is shown together with a frequency distribution (yellow) of upper critical temperature limits for 77 anurans based on data in the supplementary materials of Sunday et al. [82]. Vertical lines show the temperatures at which Andre et al. [78] found 95% mortality (red) and 50% mortality (cyan) in *Rana muscosa*.

*Batrachochytrium dendrobatidis* (Bd) has an optimum growth rate in vitro between 17–25 °C [65] (see Figure 2). Whilst the optimum temperature varies between isolates from different climatic zones, the upper critical limit for fungal growth is less than 29 °C, which ultimately defines the maximum distribution of the pathogen [13,66,67]. Most frogs, other than cold-adapted species, have maximum critical temperatures in excess of 32 °C [68] (see Figure 2) suggesting that high temperatures may enable frogs to clear infections. There is some evidence from tropical areas that some frog species persist with endemic Bd at lower, warmer altitudes but not at higher, cooler elevations [69], and that Bd prevalence is correlated with altitude [70,71].

As with all physiological processes in ectotherms, the immune function in amphibians is strongly influenced by temperature [72]. Numerous studies have found that the performance of both innate [73,74,75] and adaptive [76] immune responses are reduced at low temperatures. Rapid changes in temperature have also been found to have a detrimental effect on immune function [74,77]. Within the thermal range of Bd, it is likely that the temperature-dependent variation in a host’s immune efficacy is relatively more important for determining infection outcomes than the corresponding temperature-dependent variation in Bd growth rates (see Figure 2). For example, Andre et al. [78] found that the mortality of post-metamorphic mountain yellow-legged frogs (*Rana muscosa*) exposed to Bd was 95% at 17 °C, but only 50% at 22 °C. Similarly, Sonn et al. [79] found a positive relationship between temperature and survival from Bd infection in Northern cricket frogs (*Acris crepitans*) and Murphy et al. [80] found higher survival of Bd-infected boreal toads *Anaxyrus boreas boreas* at 18 °C than at 15 °C.

**Figure 3:**
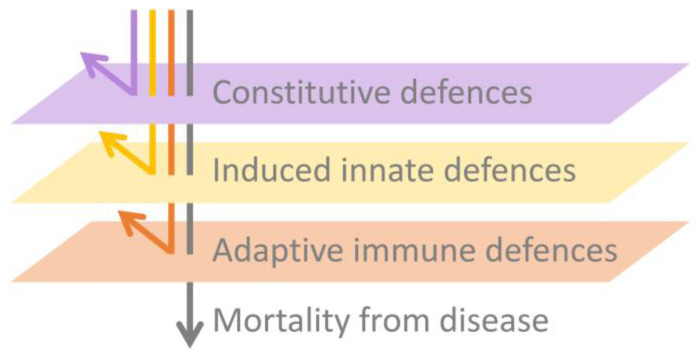
Schematic depicting the layered nature of the immune responses to infection in simplified terms. Robust constitutive defences are the most efficient and effective way to combat a pathogen because they can inhibit infection. Induced innate defences are the second line of defence against pathogens. They can rapidly arrest infection processes or reduce pathogen growth rates, buying survival time. Adaptive immune defences are initially slower to develop in a naively infected host but can be highly effective in providing a targeted defence against pathogens if the host survives the initial infection long enough. If the combined effect of these layers of defence is inadequate, then the pathogen will continue to replicate and the host will eventually die due to overwhelming pathogen burdens, if not before this, due to immunopathology.

**Figure 4:**
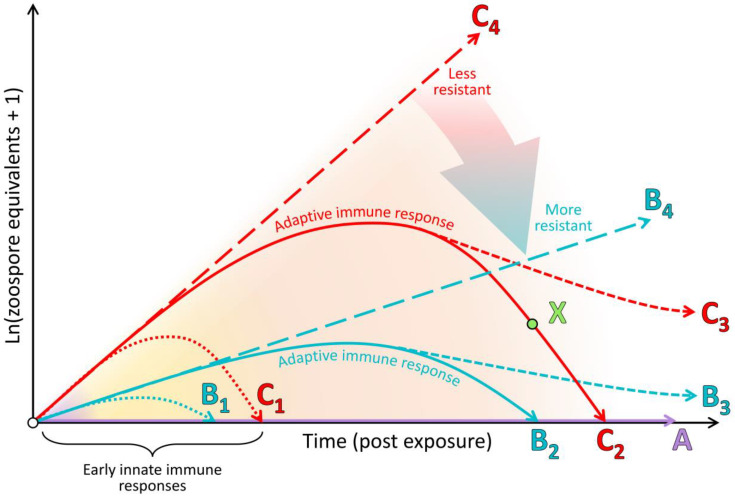
Schematic depicting the expected infection load trajectories against time post exposure and infection outcomes associated with different types of immune responses. Type A individuals (purple) possess fully effective constitutive defences, are completely resistant and do not develop infection. Type B individuals (cyan) have partially effective constitutive defences and induced innate defences, reducing their overall pathogen growth rate throughout the course of the infection (demonstrating greater resistance than type C individuals (red)). Type B individuals with sufficiently effective early innate immune responses (corresponding to the period depicted by the yellow background band) may either (1) recover (B_1_), or (2) survive long enough to develop an adaptive immune response (orange background band), from which they might then (3) recover (B_2_) or (4) remain infected (B_3_). (5) Finally, if their adaptive immune response is ineffectual, their infection trajectory may continue to increase (B_4_) until they succumb to the infection. Type C individuals do not possess effective constitutive defences; therefore, their native infection trajectory follows an exponential pathogen growth rate. If and when their induced innate and/or adaptive responses occur, similarly to type B, they may depart from this trajectory and either recover or remain infected (C_1–3_). Alternatively, if the immune responses are entirely ineffective, the infection burden will increase exponentially until the host succumbs to infection (C_4_). Importantly, individuals may die from infection at any point during any of the above-described infection trajectories due to immunopathology (e.g., it is entirely possible for individuals of type C to die at point X, despite a declining infection load).

In this paper, we expand beyond our previous detailed review [83] by presenting an integrated synthesis of the observed amphibian post-metamorphic immunological responses and the corresponding outcomes of infection with Bd. We use a simplified immunological framework where the various immune components (constitutive, induced innate and adaptive) form sequential layers of defence against Bd (see Figure 3). These layers of defence are presented according to the timing of their greatest effect in reducing pathogen growth rate or corresponding infection burden following exposure to Bd (see Figure 4). Although we considered research pertaining to infections caused by both Bd and Bsal, most of the literature to date relates to Bd. We support our synthesis with inference from the broader amphibian and mammalian immunology literature and highlight recent findings and future directions.

We commence with an overview of the amphibian constitutive defences, which are of themselves often sufficient to entirely prevent infection by Bd. We follow this with a summary of induced innate immune responses, highlighting a current general lack of knowledge surrounding this critical stage for pathogen recognition and immune activation. We discuss fungal virulence factors and intracellular fungal localisation as possible contributors to fungal evasion of the induced innate immune response. Importantly, there is little current evidence that the early innate responses induced within this stage are by themselves sufficient to halt the infectious process and eliminate the pathogen. We finish by focusing on the adaptive immune response and the alternative infection outcomes of (1) survival following indefinite pathogen suppression, or elimination and recovery, or (2) mortality following lymphocyte immunosuppression and the ensuing ineffectual inflammatory cascade, resulting in immunopathology and systemic metabolic disruption.

## 2. Constitutive Defences Are Sufficient to Prevent Infection in Many Cases

The skin is the first line of defence against pathogen invasion [84,85] and relative to other vertebrates, plays a unique and expanded role in amphibian physiology. Amphibian skin has roles in camouflage, protection from oxidative stress and UV irradiation, predator and pathogen defence, respiration, osmoregulation, thermoregulation, and reproduction (via chemical signalling) [60,86,87,88]. Immunologically speaking, amphibian skin presents a robust barrier of physical, chemical and biological defences that are always present (constitutive) [56], although some can be induced in response to infection (for example, the gene encoding the antimicrobial peptide preprocaerulein precursor is up-regulated in the spleen 7 days post infection [89]). Most superficially, a mucus layer produced by dermal mucous glands covers the skin surface (see Figure 1), promoting water conservation and providing a medium for gas exchange [90]. The mucus also provides a physical and chemical barrier to pathogen invasion and contains (1) symbiotic microbiota and their antimicrobial secretions and (2) defensive enzymes, mucosal antibodies and AMPs produced continuously by the amphibian host (Figure 1) [91].

Beneath this layer of mucus, the skin presents a further physical barrier to invading pathogens via one or more layers of keratinised squamous epithelial cells linked by cellular junctions (Figure 1; reviewed by [85]). In comparison with the more keratinised mammalian skin, amphibian skin has greater permeability to water and ions via cellular junctions (reviewed by [45]). As an additional form of defence, skin cells continuously proliferate from a basal epidermal layer and migrate towards the skin surface, whereupon they are intermittently shed, contributing to physically removing microbiota, including any pathogens present on or within the shed layer [92]. Studies involving functional depletion of microbial communities and host-produced AMPs have confirmed that in many species, these constitutive defences are themselves sufficient to prevent infection by Bd (see type A in Figure 4) [93,94,95]. In other cases, partially effective constitutive defences may help to suppress the pathogen growth rate over the course of the infection (i.e., lowering the infection trajectory from the long-dashed line of type C to that of type B in Figure 4).

### 2.1. Skin Microbiota

The skin surface and associated mucus, with its supply of oxygen, water and nutrients, provides an ideal medium for epibiotic microbial growth [88]. Microbiota, predominantly bacteria and fungi, have been demonstrated to form diverse and complex assemblages on amphibian skin (Figure 1) [96] and are thought to play a substantial role in modulating Bd infection processes [95,97,98]. They can do this via (1) directly competing with pathogenic microbes for nutrients [99]; (2) secreting antimicrobial substances with pathogen-repellent and growth-inhibiting properties [100,101,102]; (3) functionally changing the host immune response, including potential interactions with host-produced AMPs [103,104]; (4) contributing opportunistically to pathogenesis (Figure 1E) [105]. Characteristics of microbial communities that are likely associated with preventing Bd infection include the relative or absolute abundance of certain microbial species and their capacity to secrete anti-Bd metabolites (such as *Janthinobacterium lividum* and its secreted metabolite violacein) [102,106] (see [107] for a comprehensive database), the overall microbial species diversity and the community structure [104]. Probiotic therapeutic approaches, involving the application of single bacterial species and their secreted compounds [102], or consortia of bacterial species [108,109], are currently under investigation for use as disease mitigation strategies for wild populations in situ [97].

### 2.2. Antimicrobial Peptides, Defensive Enzymes and Natural Mucosal Antibodies

Amphibians constitutively produce a range of chemical defence compounds that they secrete at the skin’s surface to deter predators and/or protect against invading pathogens [48]. These include toxins, small cationic hydrophobic antimicrobial peptides (AMPs) (e.g., steroids, alkaloids and biogenic amines) [110,111], enzymes with defensive properties (e.g., lysozyme) [91,112] and mucosal immunoglobulins (e.g., natural antibodies) [113]. The amphibian AMP repertoire is remarkable amongst vertebrates [48,49], with over 1000 unique peptides and genes having been identified to date [114,115] (reviewed by [85]). The AMPs produced within the granular glands of various amphibian species have been found to be variously important for resistance against Bd [93,94,116]. Characteristics influencing this role in resisting Bd include the fungal-inhibitory properties of individual peptides (as tested in vitro), the diversity of peptides released, susceptibility to degradation by proteases and the resulting AMP concentration at the skin’s surface [117,118,119]. In contrast, the relative importance of natural antibodies and defensive enzymes, such as lysozyme, in the defence against Bd is currently unclear [91,113].

### 2.3. Skin Sloughing

Under natural conditions, amphibians periodically shed their outermost skin layers to assist with proper physiological functioning [120]. This process, known as sloughing, may assist in physically reducing the infection loads of skin pathogens, including Bd, potentially resolving chytridiomycosis infection in some species [28,92,121]. At low infection loads, sloughing was found to be relatively more successful in clearing infections, whereas later in the infection process, sloughing contributed to the disruption of skin homeostasis and morbidity [28,122]. Indeed, Bd infection typically disrupts normal sloughing processes, increasing the frequency of sloughing and reducing the size and integrity of sloughed skin pieces (Figure 1) [123]. Despite the variance between phylogenetic groups, sloughing rates do not correlate with Bd-driven declines, suggesting it is not a major contributor to patterns of Bd resistance [122].

## 3. The Induced Innate Immune Response Appears Inadequate to Resolve Batrachochytrium dendrobatidis Infection

The role of the innate immune system is to rapidly detect broadly conserved molecular structures of pathogens or the cellular and molecular damage they cause and thereafter induce an inflammatory response and/or cellular autophagy at the site of infection to remove the threat. Across the chytridiomycosis literature, there is little evidence for the early up-regulation of genes related to pathogen recognition and induction of an inflammatory cascade in response to infection [89,124,125,126]. This paucity of evidence may be due to the localised nature of early infections (the pathogen may remain undetected by immune factors or nearby immune effector cells, such as dendritic cells; see Figure 1A), but may also be explained by a distinct knowledge gap surrounding innate immune responses in amphibians at sufficiently early time points [83,85]. However, there is also little evidence for any resulting innate immune leukocyte infiltration into the skin that is capable of resolving infection (Figure 1B–E) [127,128,129,130,131]. Furthermore, Bd infection processes are generally not arrested via innate immune responses alone (i.e., the infection trajectories represented by dotted lines in Figure 4 have generally not been observed). Most infections continue to escalate in burden for weeks prior to (1) the eventual mortality of the host or (2) sufficient time has elapsed for adaptive immune responses to suppress the infection burdens (solid lines in Figure 4) [132,133]. Some possible causes for this inadequate induced innate immune response include (1) the lack or shielding of conserved fungal patterns for host recognition, (2) the intracellular localisation of the pathogen contributing to immunoevasion, and corresponding but insufficient cell-autonomous immunity, and/or (3) active suppression of recognition pathways or the resulting inflammatory cascade by the pathogen until much later in the infection process. We discuss the evidence concerning these possibilities below.

### 3.1. Classical Pathogen Recognition Pathways May Be Impaired for Infections by Batrachochytrium dendrobatidis

In the ordinary process of fungal infections, pathogen-associated molecular patterns (PAMPs, such as fungal cell wall components) should be detected by the host via pattern recognition receptors (PRRs) on myeloid immune cells (macrophages, dendritic cells and monocytes; Figure 1A,B), thereby inducing cytokine production and a resultant inflammatory response. Several classes of PRRs are known to be present in amphibians, including transmembrane Toll-like receptors (TLRs) and C-type lectin receptors (CLRs). These detect extracellular pathogens or antigens on cell membranes. In addition, cytoplasmic NOD-like receptors (NLRs) and RIG-I-like receptors (RLRs) detect intracellular pathogens [134,135,136]. Of these, CLRs are thought to be the most important for general fungal pathogen recognition [137].

However, emerging pathogens, such as Bd, may not present PAMPs that are recognised by existing host PRRs [138] or the host may not possess suitable PRRs for the PAMPs that are present. For example, the Bd genome lacks gene homologues that encode important fungal cell wall polysaccharides, such as β-1,3-glucan synthases [139]. Correspondingly, homologues of the relevant C-type lectin receptor, Dectin-1, are not present in the *Xenopus laevis* genome, and may not be present in any amphibian [140]. Although chitin and mannan are common fungal cell wall components that are thought to be present in Bd, associated PRRs were not found to be up-regulated early post infection [126]. Chitin and mannan may not be sufficiently immunogenic or may instead contribute to immune shielding [139]. Only one study thus far has found putative complement genes (complement factor B and venom factor 1 associated with the alternative pathway) that were significantly up-regulated in early infection, and only in the most resistant treatment group [126]. Consistent with the general lack of evidence for up-regulated pathogen recognition pathways, there is little evidence for the up-regulation of gene homologues for effector cytokines in the early infection stages [89,124,125,126]. Conversely, it has been demonstrated that induced innate immune components are dramatically up-regulated in the late stages of infection in amphibians that eventually die from chytridiomycosis. This likely represents nonprotective immunopathology that is triggered by widespread tissue damage and/or secondary bacterial infections (Figure 1D,E) [83,126,141].

### 3.2. Intracellular Immunoevasion and Insufficiently Effective Cell-Autonomous Immunity

Initial infection by Bd involves the injection of a germ tube into deeper host epidermal cells, transferring the contents of the encysted Bd zoospore into the host cell with the associated digestion of the host cell cytoplasm (within 8–24 h; Figure 1B) [142]. This process of injection into epithelial cells may allow Bd to avoid extracellular immunosurveillance by professional antigen-presenting cells or leukocytes, such as macrophages. However, metazoans possess a more ancient form of cellular self-defence involving all host cells, known as cell-autonomous immunity [138]. The disruption of cellular membranes and compartmental concentration gradients should activate the mechanisms of cell-autonomous immunity through danger receptors and protease-activated receptors that recognise danger-associated molecular patterns (DAMPs). The infected cell responds to these insults with a variety of mechanisms, from the release of reactive oxygen species and type I/III interferons and/or other enzymes, to activation of the inflammasome and subsequent programmed cell death via caspase and other signaling pathways.

Transcriptomic studies of chytridiomycosis have found indicators of cell-autonomous immunity occurring in early infection stages. For example, several studies on different species infected with Bd have identified marked up-regulation of interferon (IFN)-induced very large GTPase gene analogues [126,141,143]. Immune GTPases are associated with multiple antimicrobial activities within host cells, including the activation of inflammasomes [144,145,146]. Furthermore, in some studies, interleukin-1β gene homologues, a common product of inflammasomes, have been markedly up-regulated in the early post-infection stages [126]. Indeed, cell death assays have identified increased terminal transferase-mediated dUTP nick end-labelling (TUNEL) and caspase 3/7 in the skin in association with increasing infection loads [147], which could be a result of programmed cell death in response to infection. However, Bd mycotoxins appear broadly cytotoxic, and Bd supernatants that can induce splenocyte apoptosis in vitro appear to activate caspase signalling pathways [148]; therefore, the observed cell death may be the result of Bd virulence factors rather than host cell-autonomous immunity. Regardless, there is currently little evidence that the death of infected host cells is efficacious in limiting infection burdens.

### 3.3. Proinflammatory Responses Are Mild or Absent, Possibly Due to Batrachochytrium dendrobatidis Immunosuppression

Even when pathogen recognition does occur, the proinflammatory cascade (e.g., cytokines) and cellular responses (leukocyte recruitment and infiltration to the site of infection) may be suppressed or prevented, possibly due to fungal virulence factors. For example, not only is there little evidence for an up-regulated cytokine response in the early infection stage in susceptible frogs [126] but putative complement pathway genes were found unexpectedly down-regulated early post infection compared with uninfected control frogs in several studies [89,125]. Young et al. [131] found lower total circulating white blood cell numbers and impaired responses for immune stimulation. The reported neutrophil–lymphocyte ratios of infected frogs have been inconsistent between studies [149,150,151]. The histopathology of infected skin from moribund frogs has revealed that leukocyte recruitment is typically lacking in infected animals, with infrequent foci of neutrophils and macrophages detected [128,129,130]. These foci are often associated with an eroded or ulcerated epidermis during the late stages of infection, possibly occurring in response to secondary bacterial infection (Figure 1E). However, in vitro studies of Bd supernatants with peritoneal leukocytes have revealed no impairment of macrophage or neutrophil functions, such as fungal recognition or phagocytosis [148]. Furthermore, in vivo, the recruitment of innate leukocytes did not appear impaired by Bd soluble factors [152]. Although still unclear, at this stage, it seems most likely that any impairment of the innate immune response may instead be linked with intracellular immunoevasion, inadequate pathogen recognition and insufficient cell-autonomous immunity.

## 4. The Adaptive Immune Response Is Compromised by Batrachochytrium dendrobatidis-Associated Immunosuppression

The adaptive immune system comprises lymphocytes with antigen specificity (T and B cells) that provide a targeted immune response to combat a pathogen (or its antigens), along with immunological memory (the response becomes more efficient with subsequent nonlethal exposures) [84]. This latter feature of the adaptive immune system underlies the concept of immunisation (vaccination). Importantly, upon initial pathogen/antigen exposure of a naïve host, the adaptive immune response is slower to develop than both the constitutive and induced innate responses. Indeed, amphibian adaptive responses are slower and less efficacious than those of mammals (e.g., a detectable pathogen-specific antibody response can take approximately 4–6 weeks to develop in amphibians [93,153,154]; see the orange background band in Figure 4). The development of this response involves initial pathogen recognition by the innate immune system and antigen presentation and co-stimulation via major histocompatibility complex (MHC) proteins [84]. Antigen binding of lymphocyte receptors in the presence of MHC co-stimulation activates antigen-selected T and B cells to clonally proliferate, differentiate into their effector forms and migrate to the site of infection. The crucial corollary of the adaptive immune response being slower to develop initially is that infected amphibians must survive for a sufficient time for the response to manifest. If the adaptive immune response is suboptimal, instead of recovering from the infection (solid lines in Figure 4), the infected animal may instead persist with suppressed infection burdens (dashed lines in Figure 4) or may die from the infection. Mortality from an infection can occur due to the damage caused by (1) exponentially increasing pathogen loads (long-dashed lines in Figure 4) or (2) immunopathology, namely, a dysregulated immune response at any point during the infection. Current evidence suggests that most amphibians whose constitutive and innate responses are insufficient to prevent or halt an infection also demonstrate poorly protective adaptive immune responses, which is likely due to Bd-associated immunosuppression. Below, we discuss evidence concerning (1) the adaptive immune system and its capacity to respond to Bd; (2) the clinically non-protective nature of this response in most amphibians; (3) Bd-associated suppression of the adaptive immune response, particularly lymphocytes; (4) immunopathology accompanying, or as a sequela of a non-protective immune response.

### 4.1. The Amphibian Adaptive Immune System Can Respond to Batrachochytrium dendrobatidis

Despite an apparently poor innate immune response to Bd, with sufficient time, it is possible to elicit signatures of a functioning pathogen-specific adaptive immune response. Two classes (I and II) of cell surface MHC proteins form a crucial bridge between the innate and adaptive immune systems. This is because naïve lymphocytes that are resident in lymphoid tissues (e.g., spleen) must be activated initially by an MHC-bound antigen with appropriate co-stimulation. With its integral role in the activation of the adaptive immune response, the heritable yet highly variable locus encoding MHC molecules is a good target for exploring the extent of positive evolutionary selection in response to disease (see Box 2) [155,156]. Nucleated somatic cells (e.g., most host cells, including epithelial cells) can express MHC class I, while MHC class II molecules are predominantly expressed by professional antigen-presenting cells (e.g., dendritic cells, B lymphocytes and macrophages; Figure 1A). After the initial activation by antigen-presenting cells expressing MHC I–antigenic complex and co-stimulatory molecules, cytotoxic T cells recognise the MHC I–antigen complex expressed by infected somatic cells. These cytotoxic T cells then initiate programmed cell death to eliminate these infected host cells (and their intracellular pathogens). MHC class II proteins (bound to antigen and associated with co-stimulatory molecules) interact with helper T cells to activate other immune effector cells, including B cells. Studies of chytridiomycosis involving laboratory and field trials have identified associations between survival and the presence and expression of specific MHC variants and supertypes or their binding conformations (particularly MHC class II) [9,157,158,159,160].

Attempts to elicit high-titre Bd-specific serum and mucosal antibody (immunoglobulin) responses have generally been successful (at least with model species, such as *Xenopus laevis*), both via repeated pathogen exposures and injections with killed Bd [91,93]. Although Bd is an intracellular pathogen, B cells, their effector form the plasma cell and the antibodies they produce are expected to be important for controlling Bd. The B cells of amphibians and early vertebrates can both phagocytose and destroy ingested pathogens, as well as present antigens to T helper cells [52]. Antibodies can directly target extracellular pathogen components for destruction, activate the classical complement cascade or tag antigens presented by infected host cells for destruction by phagocytes or cytotoxic cells. Comparatively, T-cell-mediated adaptive immune responses (both CD8 cytotoxic T cells and CD4 T helper cells) at the site of infection are expected to be the most efficient means to reduce the burdens of intracellular pathogens such as Bd. Cytotoxic T cells should directly target and kill the host cells expressing MHC I that are infected with the reproductive form of Bd (zoosporangium). T helper cells have a variety of roles, including the production of cytokines (such as interferon γ and interleukin 17), amplifying the response to intracellular pathogens and activating macrophages. However, while it is likely that T lymphocytes can be appropriately activated (from the evidence of pathogen-specific antibody production), there is less evidence for their recruitment, both systemically and at the site of infection. Infected frogs often demonstrate reductions in circulating lymphocyte numbers [131,151], and histopathology of infected skin has revealed either no lymphocytes or only occasional foci of infiltrating lymphocytes associated with ulceration [127,128,129,130].

### 4.2. The Clinical Adaptive Immune Response Is Generally Poor and Nonprotective

A variety of unsuccessful vaccination attempts in different species have demonstrated, via subsequent live pathogen exposures, that any elicited adaptive immune response appears to lack protective efficacy at the clinical scale [80,91,161,162]. Under certain circumstances, such as in particular species or with repeated priming, it may be possible for this adaptive immune response to function protectively and reduce mortality [163]. Circumstantial evidence concerning experimental animals suggests that adaptive immune memory may be involved in animals that recover from chytridiomycosis after 4–6 weeks post exposure or in animals that persist with low-level burdens indefinitely (as reported by [133,158,164,165,166]). However, examples of improved survival associated with repeated immune priming may alternatively be due to increased reactivity of the innate response, known as an innate immune memory [167]. Whether innate immune memory is contributing to improving clinical outcomes from chytridiomycosis has yet to be resolved but appears likely [168].

### 4.3. Metabolites Produced by Batrachochytrium dendrobatidis Suppress Lymphocytes (Particularly T Cells)

The results of gene expression studies regarding adaptive immune responses have been complex. Studies sampling immune-relevant tissues (skin, spleen, liver and intestine) at early post-infection time points (3–10 days) have identified mild or no evidence of the differential expression of adaptive immune-related genes [89,124,125,126,169], a finding not unexpected given the timeframe of the adaptive response. However, one study that compared responses between populations differing in susceptibility found a more robust adaptive response 4 days post exposure in the more resistant population, suggesting earlier and more effective activation [126]. Studies performed at late infection time points (after 10 days post infection and most often with moribund frogs) have typically demonstrated the dysregulation of much greater numbers of MHC and lymphocyte-associated genes. It is important to note that both immune-activating and regulatory (inhibiting) genes are typically found to be differentially expressed in immune responses. Furthermore, gene expression studies are unable to identify the pathogen specificity of any adaptive response (which could alternatively be associated with a secondary bacterial infection). On balance, the results of these studies have demonstrated the relative and unexpected down-regulation of lymphocyte-associated (especially T cell) genes, particularly at late infection time points within the skin and spleen [126,141,170].

Consistent with the gene expression evidence, in vitro assays (involving cultured splenocytes from *Xenopus laevis* and *Rana pipiens* that were enriched for activated T and B cells) have demonstrated that Bd (live or heat-killed) and its supernatants alone can reduce or inhibit lymphocyte proliferation and cause apoptosis (preferentially of T cells) [148]. When tested in vivo, Bd supernatants significantly inhibited phytohemagglutinin-induced inflammatory swelling (testing the extent of lymphocyte infiltration into the injection site) [152]. Several immunomodulatory metabolites released by Bd (but not by related non-pathogenic chytrids) have since been identified and characterised (including methylthioadenosine, kynurenine and spermidine) and confirmed to inhibit lymphocyte proliferation and cause apoptosis in vitro [171,172]. However, if adaptive immune recoveries can occur in some species, it is not yet clear what factors may mediate the extent of Bd-associated immunosuppression.

### 4.4. Immunopathology Due to Ineffective Immune Responses Likely Plays a Key Role in Morbidity

By necessity, immune processes are a double-edged sword; while they attempt to destroy and remove pathogens, this often involves the destruction of host cells and tissues (infected cells and immune effector cells) and is highly consumptive of resources, such as energy [84,173,174]. This leads to a trade-off between (1) the extent of the immune response and associated damage, (2) the damage caused by replication of the pathogen itself, and (3) the capacity of the host to maintain normal homeostasis in the face of infection. This results in the resistance/tolerance dichotomy of host responses to infectious diseases [175,176]. Is it more profitable for the host to focus on limiting infectious burdens (aiming to resolve infection entirely) or limiting pathogen-induced damage? Ordinarily, this trade-off (particularly the extent of the immune response) is carefully regulated by immune feedback loops, regulatory factors (e.g., interleukin 10, interferon γ and 5-lipoxygenase) and up-regulated mechanisms of repair [177]. With a normal pathogen, if an infection persists (and is not readily resolved through constitutive or early induced innate defences), then a pathogen-specific adaptive immune response should develop over time, with the eventual capacity to rapidly target and remove the infectious organism. If the response is insufficient to remove the pathogen and completely resolve the infection, then the host may have the capacity to tolerate continued infection burdens, becoming a tolerant reservoir (dashed lines in Figure 4). However, even hosts whose infection burdens are declining due to effective immune responses can suffer mortality due to the extreme costs incurred by immune activation (see point X in Figure 4).

In cases where the adaptive immune response is suboptimal or ineffectual, positive feedback loops within the inflammatory cascade can continue to ramp up the immune response, regardless of the extent of damage it may cause. This is known as immunopathology, i.e., excessive damage caused by the immune response. Transcriptomics studies on chytridiomycosis have revealed that animals eventually succumbing to infection demonstrate evidence of such immunopathology [83,126,141,170,178]. This immunopathology is characterised by much higher numbers and variety of dysregulated immune genes being expressed in the final stages of infection compared to less susceptible animals and unexposed control animals. Not only is this massively dysregulated immune response unable to prevent mortality but it may contribute to morbidity [29,83]. A study of the metabolic phenotype associated with infection identified that frogs moribund with chytridiomycosis demonstrate systemic and massively dysregulated cellular homeostasis, including severe disruption of cellular energy pathways (especially the tricarboxylic acid (TCA) cycle, glycolysis and anaerobic fermentation), as well as biosynthetic and degradation pathways [29,179]. Of particular interest, alpha-ketoglutarate (AKG) and glutamate (enzymatically linked metabolites) were both severely depleted in late-stage infections [29]. Alpha-ketoglutarate has recently emerged as a “master regulatory metabolite” and is central to numerous important physiological pathways, including the TCA cycle [180]. Alpha-ketoglutarate is also a crucial energy precursor for rapidly dividing cells, particularly immune cells, and as such, has been called the “immune nutrient factor” [181]. The overwhelming immunopathology of late-stage infection [126] could be driving extreme utilisation of AKG and glutamate [29], resulting in failure of the TCA cycle and associated biosynthetic pathways [182] and leading to the observed clinical signs of extreme weakness, lethargy and mortality.

## 5. Conclusions and Areas for Further Research

In this paper, we provided a synthesis of the current understanding of the amphibian host immune response to chytridiomycosis (specifically Bd). Our focus on the layered nature of the immune response (comprising constitutive, induced innate and adaptive immune components; Figure 3) highlighted the importance of understanding, for amphibians of interest, which elements of the response are or are not functioning efficiently and how these result in differing infection outcomes (Figure 4). Our synthesis identified that many amphibians are likely resistant to Bd infection through the efficacy of their constitutive defences alone (the always present physical, chemical and cellular barriers preventing infection, including the remarkably diverse amphibian AMP repertoire and symbiotic microbial communities). Even where these constitutive defences cannot entirely prevent infection, they likely reduce pathogen growth rates and hence lower infection trajectories (Figure 4). Our examination of research pertaining to the induced innate immune response revealed some important knowledge gaps. While there is apparently little evidence for (1) early activation of the induced innate immune response and (2) any resulting innate leukocyte cellular infiltration to the site of infection, it is not yet clear why the innate system is not responding as expected. We explored several possible reasons for this inadequate response, including poor activation of pathogen recognition pathways, intracellular localisation, insufficient cell-autonomous immunity and Bd-associated suppression. This is clearly an area that needs further research. In contrast, research pertaining to the adaptive immune response paints a clearer picture of why amphibians often succumb to mortality due to chytridiomycosis. While the amphibian adaptive immune system generally has the capacity to respond to Bd, under normal infection conditions, this response appears to be nonprotective due to the Bd-associated immunosuppression of lymphocytes, particularly T-cell-mediated immunity. It is not yet clear what factors, if any, may mediate the extent of this Bd-associated immunosuppression. With continued infection, an ineffective immune response has been observed to result in immunopathology, which likely contributes to morbidity.

We list some promising areas for future research below:

- Prevention is the best cure, and mitigation strategies aimed at enhancing constitutive defences should remain a high priority for populations declining in situ [97].
- Comparative kinetic analyses of the early innate immune response (e.g., especially 8–72 h post exposure) using single-cell transcriptomics of localised infected skin versus uninfected skin may help to characterise the dynamic expression of immune-related genes and factors through time.
- The role and efficacy of cell-autonomous immunity should be explored further, particularly with regard to interferon-induced GTPases, the inflammasome and whether cell death pathways are associated with host defence or Bd virulence.
- The role and potential efficacy of immune memory (both innate and adaptive) should be examined, particularly with regard to amphibian populations in the wild. Habitats that experience temperature fluctuations may permit repeated nonlethal exposures to Bd, potentially enhancing immune memory. Work should also continue to explore whether long-term immunity can be induced in long-lived species released into the wild.
- The causes and consequences of immunopathology in contributing to morbidity and mortality should be explored further, including the role of systemic metabolic dysregulation in late infection [29,83].
- Most work to date has focused on adult amphibians, although metamorphs have frequently been identified as being a more susceptible life stage [128,183]. Improved understanding of the factors involved in determining metamorph survival and the role of different immune components in survival would be beneficial.
- Our understanding of the immune response to Bsal is still preliminary and would benefit from identifying the similarities and differences between Bd and Bsal immune responses (reviewed by [23]).
- Immunogenetic studies on the MHC locus have revealed promising results concerning the capacity for amphibians to evolve resistance to infection [9]. Nevertheless, MHC provides only part of the story with respect to heritable changes, and genome-wide association studies may provide a more complete picture [157].
- The effects of temperature on pathogen distribution and growth rates have been relatively well studied; however, the effect of temperature on the immune response to Bd is extremely important but is currently less well understood [89,133] (reviewed by [23]).
- The increasing availability of sequenced amphibian genomes [140] and technical immunological advances is likely to open up a wealth of new approaches for characterising the immune response (e.g., nanobodies and aptamers [184]).
